# Effects of bariatric surgery on inspiratory muscle strength

**DOI:** 10.1186/s40064-015-1088-2

**Published:** 2015-07-07

**Authors:** Sjaak Pouwels, Marieke Kools-Aarts, Mohammed Said, Joep A W Teijink, Frank W J M Smeenk, Simon W Nienhuijs

**Affiliations:** Department of Surgery, Catharina Hospital, Michelangelolaan 2, P.O. Box 1350, 5602 ZA Eindhoven, The Netherlands; Department of Epidemiology, CAPHRI Research School, Maastricht University, Maastricht, The Netherlands; Department of Respiratory Medicine, Catharina Hospital, Eindhoven, The Netherlands

**Keywords:** Bariatric surgery, Respiratory muscle strength, Weight loss, Respiratory physiology

## Abstract

**Background:**

The respiratory function is affected by obesity due to an increased deposition of fat on the chest wall. The objective of this study was to investigate the strength of the inspiratory respiratory muscles of obese individuals and the possible influence of bariatric surgery on it by measuring the maximum inspiratory pressure (MIP).

**Methods:**

Patients referred to a bariatric centre between the 3rd of October 2011 and the 3rd of May 2012 were screened preoperatively by a multidisciplinary team. Their MIP was measured at screening and 3, 6 and 9 months postoperative. In case of a preoperative MIP lower than 70% of predicted pressure training was provided supervised by a physiotherapist.

**Results:**

The mean age of 124 included patients was 42.9 ± 11.0 years and mean BMI was 43.1 ± 5.2 kg/m^2^. The mean predicted MIP preoperatively was 127 ± 31 in cm H_2_O and the mean measured MIP was 102 ± 24 in cm H_2_O. Three patients (2.4%) received training. Three months after surgery the MIP was 76 ± 26 cm H_2_O, after 6 months 82 ± 28 cm H_2_O and after 9 months 86 ± 28 cm H_2_O. All postoperative measurements were significant lower than preoperatively (P < 0.05). The only influencing factor for the preoperative MIP was age (p = 0.014).

**Conclusion:**

The preoperative MIP values were significantly lower than the predicted MIP values, probably due to altered respiratory mechanics.

## Background

Obesity is a chronic disease characterised by an excessive accumulation of body fat and causes damage to various body functions, such as cardiovascular, musculoskeletal, and metabolic functions amongst others (WHO 2000).

Also the respiratory function is affected by obesity due to an increased deposition of fat on the chest wall. This causes a reduction in thoracic compliance, lung volumes and capacities (Hamoui et al. 2006; Sarikaya et al. 2003; Weiner et al. 1998). Respiratory dysfunction in this patient population can occur due to changes in the relationship between lung, chest wall and diaphragm, causing an impairment of respiratory mechanics and also changes in gas exchange (Magnani and Cataneo 2007).

Several studies have demonstrated that weight loss due to bariatric surgery has resulted in a huge improvement in some functions, such as a decrease in haemoglobin and haematocrit (Zavorsky et al. 2008), decreased heart rate and oxygen consumption (Zavorsky et al. 2008) and a reduction in insulin resistance. Bariatric surgery also showed an improved lung function with increased forced vital capacity (FVC) (Zavorsky et al. 2007; Davila-Cervantes et al. 2004), forced expiratory volume in one second (FEV1), and improved alveolar-capillary diffusion capacity (Zavorsky et al. 2008) and an improvement in gas exchange (Zavorsky et al. 2007; Davila-Cervantes et al. 2004). However, studies on the respiratory muscle function of the obese population (before and after bariatric surgery) show conflicting results (Magnani and Cataneo 2007; Wadstrom et al. 1991a; Kelly et al. 1988; Sampson and Grassino 1983; Black and Hyatt 1969).

Thus, the objective of this study was to investigate the strength of the respiratory muscles of obese individuals (before and after bariatric surgery) by measuring the maximum inspiratory pressure (MIP). Also we have investigated the influence of patient related factors and type of operation (sleeve gastrectomy or gastric bypass) on the MIP before and after bariatric surgery. Furthermore, the association of the body mass index (BMI) and the presence of comorbidities with the respiratory muscle strength were reviewed.

## Methods

Informed consent was obtained of all patients included.

### Patient population

Patients were referred to a bariatric centre by their general practitioner or other physicians. A multidisciplinary team screened all patients preoperatively, consisting of a physician assistant, a psychologist, a nutritionist and a surgeon. Patients were eligible for surgery if they had a BMI of 40 kg/m^2^ or higher, or a BMI between 35 and 40 kg/m^2^ with significant co-morbidities, with serious attempts to lose weight in the past. Co-morbidities were considered significant when medication had to be used, or if continuous positive airway pressure (CPAP) had to be used in case of obstructive sleep apnoea (OSAS). The indication for a bariatric procedure was made in a multidisciplinary consultation, taking into account the patients’ preference, age, polypharmacy, reflux complaints, body composition in relation to BMI and bowel diseases. Patients with pre-existing pulmonary and/or neuromuscular diseases were excluded from this study.

### Respiratory muscle function and MIP assessment

Respiratory muscle strength was assessed by measuring the maximal inspiratory pressure (MIP) with a digital mouth pressure meter (±300 cm H_2_O). The manometer (Micro RPM^®^, Carefusion, USA) was calibrated in accordance with the manufacturer’s recommendations (Celli and MacNee 2004).

The maximal static respiratory pressure generated in the mouth, after complete inhalation and exhalation, carries out the measurement of respiratory muscle strength, divided in the MIP and maximal expiratory pressure (MEP), which are indicative of the strength of the inspiratory and expiratory muscle groups (Costa et al. 2010). MIP is a measure of inspiratory muscle strength (which is representative for the function of the diaphragm), whereas MEP measures the strength of the abdominal and intercostal muscles (Barbalho-Moulim et al. 2011a, 2013; Pazzianotto-Forti et al. 2012).

The assessment of the respiratory muscle strength took place before bariatric surgery and 3, 6 and 9 months postoperatively by an independent and trained physiotherapist using the ATS/ERS guidelines (Celli and MacNee 2004; American Thoracic Society 1995). The MIP values were compared to each other and to the normal values adjusted for their sex and age (Wilson et al. 1984).

When the actual preoperative measured MIP was ≤70% of the predicted MIP, patients were asked to conduct respiratory function training prior to surgery. The protocol for training of the respiratory function was adapted from a study of (Hulzebos et al. 2006).

### Data collection

Data were collected prospectively using an online registry (Patients Outcome Measurement Tool). The patients who got approval between the 3rd of October 2011 and the 3rd of May 2012 were included in this study as during this period the inspiratory muscle function was measured.

Data of interest were MIP values, patients’ characteristics, operative details, hospital stay and evolution on weight and comorbidities. Furthermore were included the postoperative pulmonary complications defined as pulmonary infections as determined by the physician, thromboembolic events, and respiratory distress resulting into additional care.

### Statistical analysis

Continuous variables were presented as mean ± standard deviation (SD). Categorical variables were presented as frequency with percentages. The Shapiro–Wilk test was used to test each variable for normality. Student’s t test for independent groups or the Mann–Whitney U test was used to compare the MIP values in the preoperative period and changes in MIP values over time, depending on the normality or non-normality of the data distribution. To compare the MIP values at different times (preoperatively, and after 3, 6 and 9 months after surgery), repeated measures ANOVA was used. To assess postoperative MIP values (and the difference with preoperative MIP values) between patients who had a sleeve gastrectomy and gastric bypass, the two-repeated ANOVA was used.

The Pearson correlation coefficient was used to determine the correlation between de following parameters: preoperative BMI and preoperative MIP; Prevalence of OSAS and preoperative MIP; weight loss 3 months after surgery and MIP 3 months after surgery; weight loss 9 months after surgery and MIP 9 months after surgery; smoking and MIP values (preoperative and after 3, 6 and 9 months); gender and preoperative MIP and age and preoperative MIP.

In all tests, values of p < 0.05 were considered statistically significant. Statistical Package for Social Sciences (SPSS, Chicago, IL, USA Version 20.0) was used to prepare the database and for statistical analysis.

## Results

### Patients and procedures

All 124 patients completed the 9 months follow-up and were all available for the final analysis. The majority of the included 124 patients were female (n = 106). The mean age was 42.9 ± 11.0 years and mean BMI was 43.1 ± 5.2 kg/m^2^. Related comorbidities encountered were diabetes (20.2%), OSAS (13.7%), hypertension (33.9%) and dyslipidemia (20.2%). Among the patients 17 (13.7%) were tobacco users and 11 (8.9%) used alcohol. Half the patients underwent a sleeve gastrectomy (n = 60; 49.2%) and the other half received a gastric bypass (n = 64; 50.8%). These interventions resulted into a weight loss of 18 ± 26 kg (13 ± 21% TWL) and 51 ± 29 kg (41 ± 23% TWL) respectively at 3 months and 1 year postoperative. No pulmonary complications as defined in the methods section were seen postoperatively.

### Maximal inspiratory pressures

In the preoperative period, the mean predicted MIP was 127 ± 31 in cm H_2_O and the mean measured MIP was 102 ± 24 in cm H_2_O (Table 1). Based on these results, only three patients were asked to train their respiratory function, because they had a preoperative measured MIP value <70% of predicted. Three months after surgery the MIP was 76 ± 26 cm H_2_O, after 6 months the MIP was 82 ± 28 cm H_2_O and after 9 months 86 ± 28 cm H_2_O. At all three measure moments this decrease was significant compared to the preoperative value (P = 0.01).

**Table 1:** Maximal inspiratory pressures of obese patients before and after a bariatric procedure

Predicted MIP^a^ (n = 124)	Actual MIP preoperative^a^ (n = 124)	After 3 months^a^ (n = 124)	After 6 months^a^ (n = 124)	After 9 months^a^ (n = 124)
127 ± 31	102 ± 24^A^	76 ± 26^B,C^	82 ± 28^B,C^	86 ± 28^B,C^

^a^In cm H_2_O.

^A^Significant difference compared with Predicted MIP (p = 0.019).

^B^Significant difference compared with Actual MIP (p = 0.01).

^C^No significant difference between 3, 6 and 9 month values (p = 0.273).

### Sleeve gastrectomy versus gastric bypass

The preoperative MIP, the MIP 3 months after surgery, 6 months after surgery and 9 months after surgery did not significantly differ between patients who had a sleeve gastrectomy compared to a gastric bypass (P = 0.590, P = 0.592, P = 0.165 and P = 0.895 respectively).

### Changes in MIP values over time

Table 2 gives an overview of the mean MIP changes between different time points. There was a significant difference between the mean changes in MIP after 3 months and 6 months (respectively −26.65 ± 22.30 and 6.03 ± 19.99 cm H_2_O, p = 0.001) and there was a significant difference in the mean changes in MIP after 3 and after 9 months (respectively −26.65 ± 22.30 and 4.07 ± 11.98 cm H_2_O, p = 0.001).

**Table 2:** Mean MIP changes (± SD) in cm H_2_O between different time points

	MIP after 3 months	MIP after 6 months	MIP after 9 months
Compared with			
Actual Preop MIP	−26.65 ± 22.30	−20.61 ± 20.58	−16.54 ± 21.08
MIP after 3 months		6.03 ± 19.99	10.11 ± 20.65
MIP after 6 months			4.07 ± 11.98
MIP after 9 months			

### Correlations between different variables

Table 3 gives an overview of the correlations investigated. There was no linear correlation between the preoperative BMI and preoperative MIP (r = −0.045, p = 0.619), the prevalence of OSAS and the preoperative MIP (r = −0.025, p = 0.779), weight loss 3 months after surgery and MIP 3 months after surgery (r = −0.187, p = 0.008), weight loss 9 months after surgery and MIP 9 months after surgery (r = −0.075, p = 0.072) and smoking and the MIP values preoperative (r = −0.003, p = 0.976) and 3, 6 and 9 months postoperative (r = 0.070, p = 0.439; r = 0.054, p = 0.552; and r = 0.014, p = 0.877 respectively). The correlation between gender and the preoperative MIP showed a trend towards significance (r = −0.155, p = 0.085). The only (negative) linear correlation found was between age and the preoperative MIP (r = −0.220, p = 0.014).

**Table 3:** Overview of the correlations between different variables (n = 124 patients)

Variable 1	Variable 2	Correlation coefficient	P value
Preoperative BMI	Preoperative MIP	r = −0.045	p = 0.619
Prevalence of OSAS	Preoperative MIP	r = −0.025	p = 0.779
Weight loss 3 months after surgery	MIP 3 months after surgery	r = −0.075	p = 0.408
Weight loss 9 months after surgery	MIP 9 months after surgery	r = −0.187	p = 0.072
Smoking	MIP preoperative MIP after 3 months MIP after 6 months MIP after 9 months	r = −0.003 r = 0.070 r = 0.054 r = 0.014	p = 0.976 p = 0.439 p = 0.552 p = 0.877
Gender	Preoperative MIP	r = −0.155	p = 0.085
Age	Preoperative MIP	r = −0.220	p = 0.014

## Discussion

The actual measured MIP was significantly lower than the predicted values in the present study. The MIP at 3, 6 and 9 months after bariatric surgery were decreased significantly compared with the preoperative MIP. The only (negative) linear correlation found was between the age and the preoperative MIP, which is corresponding with earlier studies on this subject (Magnani and Cataneo 2007; Wilson et al. 1984).

Obesity has a detrimental effect on the pulmonary physiology, including respiratory mechanics, airway resistance, respiratory muscle function, lung volume, work of breathing (WOB) and gas exchange (Koenig 2001). Morbidly obese patients present with increased metabolic demands due to a deposition of fat in the chest wall, which results in an increased mass to move during breaths and therefore a higher WOB. This elevated WOB results in reduced chest wall compliance. Also there is an elevation of the diaphragm, which (upon contracting) acts under pressure of a distended abdomen (Wei and Wu 2012; Parreira et al. 2012; Jubber 2004).

This ‘overload’ triggers a variety of mechanisms in the activity of the respiratory muscles and causes a long term training effect, which can increase muscle strength (Magnani and Cataneo 2007; Wei and Wu 2012; Parreira et al. 2012). It is believed that this muscle strength decreases when patients develop a condition such as OSAS (Magnani and Cataneo 2007). In the postoperative period, weight reduction may promote an improvement in respiratory mechanics and compliance, improving the efficiency of the respiratory muscles (Weiner et al. 1998). In the present study, the MIP after was decreased 3 months after surgery, but at 6 and 9 months after surgery it increased. This is the effect of weight loss, which reduces the earlier mentioned ‘overload’ and therefore creates a new setpoint to which the respiratory muscle strength has to adjust. This could be an explanation for the fact that the MIP decreased 3 months after surgery in our study. However we found a trend towards a negative correlation between weight loss 9 months after surgery and the MIP 9 months after surgery (p = 0.072), which indicates a decrease in respiratory muscle strength when the weight loss increases. The explanation for this matter lies in the earlier mentioned ‘overload’, which vanishes after successful bariatric surgery. Whether this earlier mentioned new ‘setpoint is due to an improvement in diaphragm muscle function or a change lung compliance is unknown.

Also animal studies in rabbits show that chronically increased intra-abdominal pressure induce several histological and cellular changes in composition of greater abdominal muscles, especially the rectus abdominis and diaphragm muscle (Kotidis et al. 2011, 2012; Papavramidis et al. 2011, 2012). Changes in muscle fiber composition of the muscles were observed, an increased ratio of type II muscle fibers that are mainly anaerobically active (Kotidis et al. 2011, 2012; Papavramidis et al. 2011, 2012).

Studies investigating indices of respiratory muscle strength in obese patients and comparing them with eutrophic individuals or comparing them with normal values showed no consistent results (Sarikaya et al. 2003; Magnani and Cataneo 2007; Kelly et al. 1988). Kelly et al. (1988) found no significant results in MIP values among obese individuals (with an average of 183% of the predicted weight) and individuals with an average of 99% of the weight. Sarikaya et al. (2003) showed a significantly reduced MIP in obese individuals with no significant difference compared with eutrophic individuals. Magnani and Cataneo (2007) found that the MIP was within normal values for age and gender in 99 obese individuals (23 men and 76 women). There was no significant difference between different BMI groups (35–40, 40–45, 45–50 and ≥50 kg/m^2^) (Magnani and Cataneo 2007).

In the literature investigating the effect of bariatric surgery on respiratory pressures, there are also conflicting results. Weiner et al. (1998) showed that maximum respiratory pressures increased 6 months after bariatric surgery when compared with the preoperative values in 21 obese patients.

Parreira et al. (2012) assessed the MIP of 30 morbidly obese patients (24 women and 6 men) preoperatively and 1 and 6 months after surgery. They found no significant difference in MIP 1 and 6 months after surgery compared to the preoperative values (preoperative MIP: 96 ± 35; after 1 month: 100 ± 38; after 6 months: 104 ± 33 cm H_2_O) (Parreira et al. 2012). When comparing the MIP values of 17 individuals 36 months after surgery, they found a significantly increased MIP (121 ± 35 cm H_2_O) compared to the preoperative MIP (96 ± 35 cm H_2_O) (Parreira et al. 2012).

Cherniack and Guenter (1961) found that obese individuals have inefficient respiratory muscles due to reduced chest wall compliance or lower lung volume. Also the MIP was lower than the predicted value (Cherniack and Guenter 1961). Wadstrom et al. (1991b) found that, despite a weight loss of 18% and improvement of lung volumes, the included obese individuals showed no significant change in respiratory muscle strength.

Barbalho-Moulim et al. (2011a) randomised 32 obese women undergoing elective open bariatric surgery to either an inspiratory muscle-training group or usual care group. Compared to the preoperative values, the MIP decreased significantly in both groups. However the reduction in MIP was 28% in the inspiratory muscle training group en 47% in the usual care group (Barbalho-Moulim et al. 2011a). In a different patient cohort (24 obese women scheduled for Roux en-Y gastric bypass), Barbalho-Moulim et al. (2013) found that compared to preoperative MIP values, the MIP 1 year after bariatric surgery significantly decreased (preoperative: 78.75 ± 20.07 cm H_2_O; 1 year after surgery: 69.17 ± 18.86 cm H_2_O, p = 0.0183) (Barbalho-Moulim et al. 2013).

Various studies have examined the correlation between MIP and body composition but found different results. Vincken et al. (1987) found that body composition did not contribute in explaining MIP variability. Enright et al. (2004) reported that weight and waist circumference was negatively related to the MIP. Carpenter et al. (1999) showed that individuals with a higher BMI had a lower MIP. We have not found a significant correlation between body composition and MIP. A possible explanation could be the difference in sample size, than the earlier mentioned studies (Vincken et al. 1987; Enright et al. 2004; Carpenter et al. 1999).

Different results were mentioned about the correlation between smoking status and MIP. Hautmann et al. (2000) demonstrated that there was no significant relation between smoking status and MIP. Leech et al. (1983) also did not found a significant relation between smoking status and MIP. Therefore Enright et al. (1994) found that smokers had a 15% lower MIP than non-/former smokers. In our study we could not find a relationship between smoking and the MIP values. A possible explanation for this matter is the small number of smokers in our study population [17 (13.7%)].

Our study has several limitations. First, our male/female equilibrium was not equal. The majority of our patients were female. Second, there is a known difference between the MIP values between males and females (Carpenter et al. 1999; Hautmann et al. 2000), which could be a confounding factor in the interpretation of the results.

The significant difference between the predictive MIP and the preoperative measured MIP was found using the equation by Wilson et al. (1984). This could imply two remarks. First, is unknown whether or not the used predictive equation is suitable for the obese population and secondly other commonly used predictive equations could influence the results (Black and Hyatt 1969; Costa et al. 2010; Neder et al. 1999; Harik-Khan et al. 1998).

The clinical relevancy of measuring the MIP prior to and after bariatric surgery is questionable. Two questions need to be answered to determine the clinical relevancy (1) How many bariatric surgical teams measure MIP routinely and (2) if the MIP is measured, what is its influence on the decision-making progress in qualification and preparation of patients for bariatric surgery?

The earlier mentioned study of Barbalho-Moulim et al. (2011a) found that inspiratory muscle training prevented a reduction in MIP postoperatively. Another study by Barbalho-Moulim et al. (2011b) compared the effect of laparoscopic bariatric surgery with open bariatric surgery on the lung function, without preoperative training of the patients. In both groups, there was a decrease in MIP postoperatively, 23% in the laparoscopic group compared to 37% in the open group (Barbalho-Moulim et al. 2011b). Both studies did not investigate the effect on postoperative (pulmonary) complications.

In the current Dutch bariatric practice, almost all bariatric interventions are performed via laparoscopic procedures and pulmonary complications are rarely seen. Also in this study no pulmonary complications were seen. Therefore in other types of surgery, per example cardio-thoracic surgery, determining the MIP is useful for clinical purposes. This because after coronary artery bypass grafting surgery, pulmonary complications are more frequently seen and therefore inspiratory muscle training (Hulzebos et al. 2006). In our opinion, based on the current body of literature, the clinical relevance of measuring MIP prior to bariatric surgery remains questionable. Therefore further research is needed to investigate the clinical usefulness of this MIP measurement, especially in patients with obesity-induced respiratory dysfunction.

## Conclusion

The preoperative MIP values were significantly lower than the predicted MIP values. Only three patients were indicated to train their respiratory function preoperatively. Also a significant decrease in maximum pressures was found 3, 6 and 9 months after bariatric surgery each compared to the preoperative measurements. A negative significant correlation was observed between age and preoperative MIP. Due to conflicting results in the current literature, the low number of pulmonary complications seen after bariatric surgery the clinical relevancy of measuring the MIP prior to and after bariatric surgery remains questionable.
